# Scholarly productivity and professional advancement of junior researchers receiving KL2, K23, or K08 awards at a large public research institution

**DOI:** 10.1017/cts.2016.22

**Published:** 2017-04-19

**Authors:** John K. Amory, Diana K. N. Louden, Christy McKinney, Joanne Rich, Stacy Long-Genovese, Mary L. Disis

**Affiliations:** 1 Departments of Medicine, University of Washington, Seattle, WA, USA; 2 Institute of Translational Health Sciences, University of Washington, Seattle, WA, USA; 3 University of Washington Libraries, University of Washington, Seattle, WA, USA; 4 Department of Oral Health Sciences, University of Washington, Seattle, WA, USA

**Keywords:** Career development, translational research, CTSA, interdisciplinary research

## Abstract

**Background:**

How the productivity and careers of KL2 scholars compare with scholars receiving individual K-awards is unknown.

**Methods:**

The productivity of KL2 scholars (n=21) at our institution was compared with that of K08 (n=34) and K23 (n=26) scholars.

**Results:**

KL2 and K23 scholars had greater productivity than K08 scholars (*p*=0.01). Professional advancement was similar among groups.

**Conclusion:**

At our institution, scholarly productivity and professional advancement did not differ by type of K-award.

## Introduction

Becoming an independently funded investigator in biomedical research is challenging [1–3]. To assist the development of junior faculty, the National Institutes of Health (NIH) has funded a series of career development or “K” awards, which provide junior faculty with significant protected time to acquire the training and experience necessary to transition into independently funded investigators. The most common awards for investigators seeking careers in translational research are the K08 for laboratory-based investigators and the K23 for investigators involved in clinical research.

In 2005, The Clinical and Translational Science Awards (CTSA) program was initiated by the NIH to support clinical and translational research in academic, biomedical research institutions. As the NIH recognized the need to maintain the pipeline of investigators involved in translational research, the CTSA included an Institutional Mentored Clinical Research Scholars program, called the KL2, to support the career paths of selected junior faculty involved in clinical and translational research. Nationally, over 50 CTSA institutions support KL2 awardees at a cost of ~45 million dollars annually [4]. Assessments of the KL2 program suggest that scholars have improved clinical research self-efficacy after participation [5] and are likely to remain active in research after completing their period of KL2 training [6]. Despite these insights, more information regarding the outcomes of KL2 scholars would be informative. In particular, how the research productivity and professional status of scholars enrolled in KL2 programs compares with junior faculty receiving translational K08 or K23 awards has not been reported.

To examine this, we compared the research output in terms of scholarly publications of all 21 KL2 scholars supported at our institution and compared them with the 60 K08 and K23 scholars awarded during the same period in a quasi-experimental, observational design. We hypothesized that KL2 scholars would have greater research productivity compared with scholars with individual K-awards. We also examined NIH grant awards to see whether funding success in terms of NIH R-awards differed between the 3 groups of K-scholars. As our institution is one of the “top 10” institutions in terms of the number of K-awards nationally [7], we felt that this information would be of interest generally as part of ongoing national efforts to build a robust translational research workforce.

## Methods

We searched the NIH RePORTER database for recipients of KL2, K08, and K23 awards at the University of Washington who began their K-award funding between 2005 and 2010. We chose this interval so that all scholars included in the analysis would have completed at least 5 years of K-support through July of 2015. To determine research productivity, the multidisciplinary database Scopus was searched for original, scholarly research publications using the scholar’s name. Reviews, case reports, editorials, and other nonresearch article types were not included. As all information was obtained using publicly available resources, scholars were not consented before the analysis.

To determine the number of disciplines represented by each publication, for each research article, the number of co-authors and the departmental affiliations of each co-author were tabulated. For each author affiliation, the individual’s department or division was categorized using a modified version of the “NIH Field of Training Classification” (see http://grants.nih.gov/training/phs2271.pdf). The funding status of the K-scholars was determined by a computer search of NIH RePORTER. Continued research activity was determined by searching Scopus, and defined as continuing to co-author at least 1 original research paper every year over the last 3 years. Promotion status and current research status and positions were determined by internet searches, which successfully determined current professional status in all cases.

### Statistical Analysis

We compared the demographic characteristics of K-scholars, grant awards, and academic status using an extended χ^2^ test. As the distributions of the number of research publications produced during K-support, numbers of co-authors, and disciplines were not normally distributed, we present the data as box plots with medians and interquartile ranges. We compared the number of research articles, co-authors, and disciplines by type of K-award using a Kruskal–Wallis analysis of variance with Wilcoxon rank-sum post hoc test. All analyses were performed on STATA version 10.0 (StataCorp LP, College Station, TX, USA). For all comparisons, an α of 0.05 was considered statistically significant.

## Results

### Scholar Characteristics

We identified 81 K-scholars who received an NIH K08, K23, or KL2 award, between 2005 and 2010, at the University of Washington. The characteristics of the K-scholars by cohort and together are shown in Table 1. There were trends toward greater male representation and a greater representation of M.D.s in the K-08 award cohort as compared with other K-awards. Overall, 86% of K-awards were received by faculty at the School of Medicine, of whom almost all were M.D.s or M.D./Ph.D.s, with Schools of Dentistry and Nursing each accounting for 5% of the total and Schools of Pharmacy, Social Work, and Public Health only having 1 K-scholar each. The percentage of non-M.D.s was slightly higher in the KL2 cohort than the other K-cohorts, but this difference was not statistically significant (*p*=0.06). Overall, representation of under-represented minorities in all programs was low at 5% of all scholars; this percentage was highest in the KL2 group at 15% (*p*=0.2).

**Table 1:** Characteristics of K-scholars at the University of Washington awarded a type of K-award (2005–2010). Data are presented as number of individuals (%)

Characteristics	KL2 (n=21)	K08 (n=34)	K23 (n=26)	Total (n=81)	*p* Value
Sex					
Male	12 (57)	24 (71)	10 (40)	46 (57)	0.06
Female	9 (43)	10 (29)	16 (60)	34 (43)	
Doctoral degree(s)					
M.D.	9 (43)	20 (59)	16 (64)	45 (56)	0.10
M.D./Ph.D.	5 (24)	11 (32)	4 (12)	19 (24)	
Ph.D.	7 (33)	3 (9)	6 (24)	16 (20)	
Discipline					
Medicine	15 (71)	32 (94)	23 (88)	69 (86)	0.18
Dentistry	1 (5)	1 (3)	2 (8)	4 (5)	
Nursing	3 (15)	0 (0)	1 (4)	4 (5)	
Pharmacy	1 (5)	0 (0)	0 (0)	1 (1)	
Social Work	1 (5)	0 (0)	0 (0)	1 (1)	
Public Health	0 (0)	1 (3)	0 (0)	1 (1)	
Medicine	15 (71)	32 (94)	23 (88)	69 (86)	0.06
Non-Medicine	6 (29)	2 (6)	3 (12)	11 (14)	
Race/ethnicity					
White	11 (52)	27 (79)	21 (80)	58 (73)	0.24
Asian	7 (33)	7 (21)	4 (16)	18 (23)	
African-American	1 (5)	0 (0)	1 (4)	2 (3)	
Hispanic	1 (5)	0 (0)	0 (0)	1 (1)	
American Indian/Alaskan Native	0 (0)	0 (0)	0 (0)	0 (0)	

### Research Productivity

**Fig. 1:**
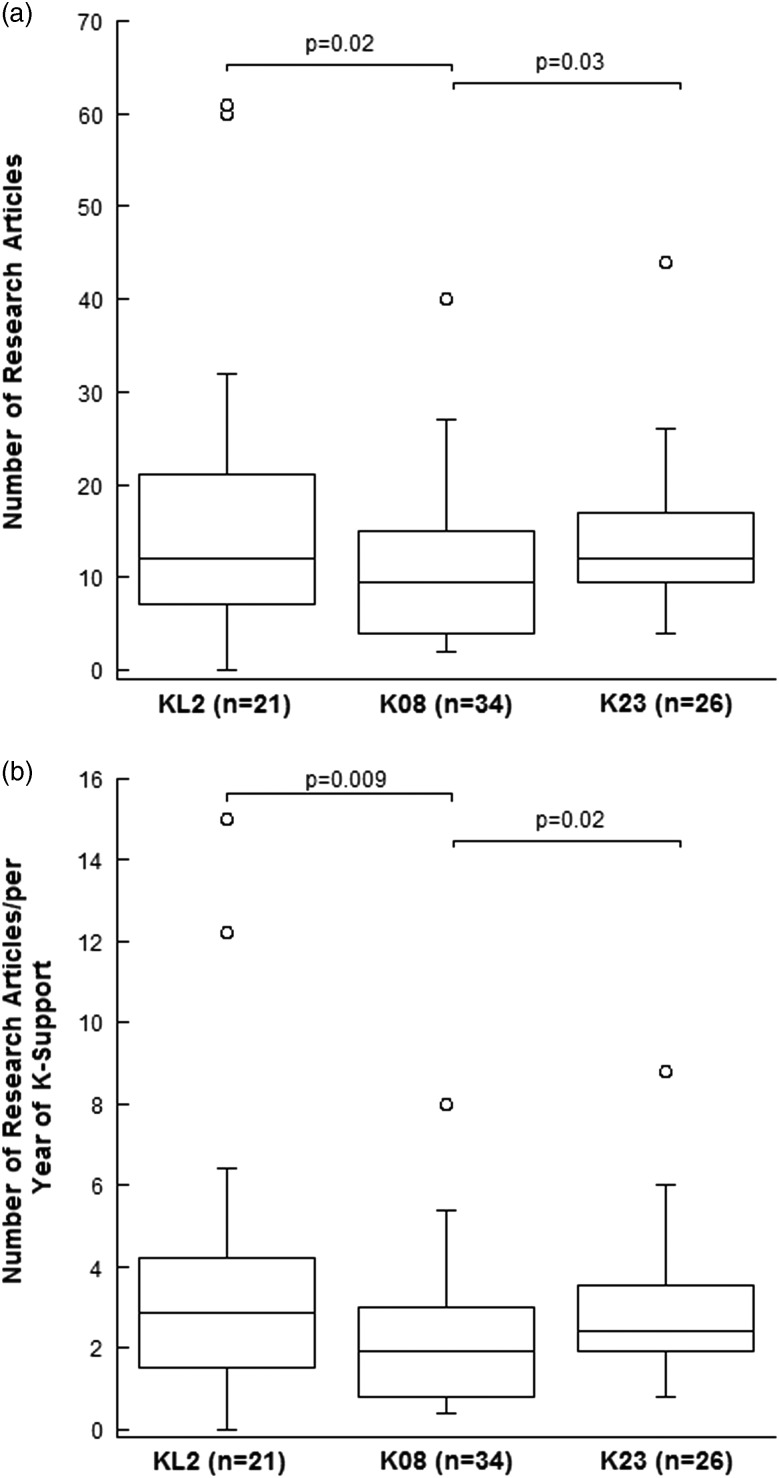
Number of research articles (*a*) and articles per year (*b*) by K-scholars at the University of Washington by K-award type (2005–2010).

The median number of peer-reviewed publications of original research during the scholar’s period of K-support is depicted in Fig. 1*a*. Scholars in the KL2 and K23 programs had slightly higher publication counts (PC) than scholars in the K08 program [median (interquartile ranges) KL2: 12 (10, 21) PC; K23: 12 (10, 18) PC; K08: 9 (4, 15) PC; *p*=0.02 for KL2 vs. K08 and *p*=0.03 for K23 vs. K08]. Similarly, the median number of publications per year (P/Y) was as follows: KL2: 3 (2, 4.2) P/Y; K23: 2.4 (2, 3.6) P/Y; K08 1.8 (0.8, 3) P/Y (*p*=0.009 for KL2 vs. K08; *p*=0.02 for KL2 vs. K23) (Fig. 1*b*). There were no statistically significant differences in research publications or publications per year between the KL2 and the K23 cohorts. Extended follow-up of theses cohorts (average follow-up 8 years) did not reveal any statistically significant differences between groups (*p*=0.34) over time. Interestingly, the median number of articles published per year decreased in the post-K funding period for all groups from an overall median of 2.4 (1.2, 3.5) P/Y to 1.8 (1.1, 2.8) P/Y during the post-K period (*p*<0.001). The median number of co-authors per article was 7.5 (5.5, 10.2) for all K scholar cohorts (*p*=0.80). There was no evidence of greater interdisciplinarity in the research of KL2 scholars, as the median number of disciplines represented per research article was 2.6 (2.3, 3.8) for all groups of K-scholars (*p*=0.63).

### Professional Advancement and Continued Research

Thirty-six percent of the scholars were awarded an NIH R-grant as principal investigator, with 26% being awarded an R01 and 10% being awarded another R-award such as an R03 or an R21 (Table 2). There was no significant difference in the receipt of R-awards by type of K-award. Overall, 41% of the scholars achieved the rank of Associate Professor during the period of study, which was similar between groups. Ninety-three percent of the K-scholars in this analysis continued to produce published research, including all 21 of the KL2-scholars. At present, 88% of the K-scholars are working in academia, 6% conducting research in other settings, and 6% are neither in academic settings nor performing research, and instead are working in private practice (Table 2). Interestingly, this represented 8%–10% of the K08 and K23 recipients, but none of the KL2 recipients.

**Table 2:** Current research, grant, and academic status of K-scholars at the University of Washington by type of K-award (2005–2010). Data are presented as number of individuals (%)

Characteristics	KL2 (n=21)	K08 (n=34)	K23 (n=26)	Total (n=81)	*p* Value
Research status					
All research*	21 (100)	31 (91)	24 (92)	75 (93)	0.58
Academic research	18 (85)	30 (88)	22 (88)	70 (88)	
Foundation/industry	3 (15)	1 (3)	1 (4)	5 (6)	
No research	0 (0)	3 (9)	2 (8)	5 (6)	
Grant status					
R01	6 (29)	10 (29)	5 (20)	21 (26)	0.50
Any R-award	7 (33)	14 (41)	8 (32)	29 (36)	
Academic status					
Associate Professor or Professor	11 (52)	16 (47)	6 (24)	33 (41)	0.10
Other	10 (48)	18 (53)	19 (76)	47 (59)	

* Defined co-authorship of more than 1 manuscript of original research yearly in Scopus over the last 3 years.

## Discussion

Given the importance of maintaining a healthy workforce of translational researchers, there has been great interest in understanding the nature of career development for junior faculty researchers [8]. Several recent publications have described career pathways for junior faculty engaged in biomedical research; however, to our knowledge, no comparison of the research productivity and professional advancement of different types of K-awardees has been published. Our study addressed this gap in knowledge. We found that research productivity and professional advancement do not appear to significantly differ by K-program at our institution. Scholars receiving K08 awards appear to have slightly lower research productivity compared with scholars in the KL2 and K23 programs, probably due to the laboratory-based research focus of these awards. In contrast, the median research productivity of the KL2 and K23 scholars is between 2 and 3 papers annually. This degree of productivity may be informative to junior faculty seeking to understand how many publications are produced by the “average” K-scholar on a yearly basis. Previous productivity analyses in the literature have found similar productivity [9], but have not focused solely on research publications, which are more germane for career development.

In contrast to our original hypothesis, the research produced by KL2 scholars does not appear to be more interdisciplinary compared with the research produced by scholars of other K-types. This lack of difference could be due to the high baseline levels of interdisciplinary research by all K-scholars at our institution, such that the KL2 program does not confer any measureable additional benefit. Alternatively, our measurement of interdisciplinarity may have lacked sensitivity. Other measures of “interdisciplinarity” have been used to understand the nature of collaboration in translational science [10], but each measure has drawbacks to interpretation, and no one measure is clearly superior. Finally, it is possible that participation in the KL2 program has no impact on the interdisciplinarity of the research produced by scholars. Given the perceived need for researchers of different types to address complex biomedical topics in the future, additional attention to ways to measure and promote interdisciplinary collaborations are needed.

It is encouraging to note the high percentage of K-scholars who remain active in research. Interestingly, the research productivity of scholars seems to be slightly reduced after their period of K-support. This appears to be largely because of decreased research productivity in the fraction of scholars who have not yet obtained independent funding after their period of K-support, and likely reflects a decrease in the amount of time available to these individuals to conduct research.

The ability of transition from K-awards to R-awards, the “K to R transition,” has been extensively studied. Jagsi *et al*. [11] found that roughly 40% of K-recipients obtained an R01 within 10 years of receiving a K-award. More recently, a comprehensive report performed at the NIH revealed that among 8229 individuals awarded a K-award between 1999 and 2008 75% of former K-awardees applied for an R01 and 41% were awarded an R01 grant [12]. We observed a slightly lower rate of receipt of R01 in our cohort; however, this is not surprising as our cohort represented included scholars who began their K-award in 2010 and have had less time to successfully compete for R-level funding.

There are some weaknesses to our analysis. First, our sample size was small as it was limited to the 81 K-scholars beginning their period of support at our institution between 2005 and 2010. Second, our data are from a single center with a high level of research accomplishment, and therefore the results may not be reflective of all research environments, especially those with fewer resources. Finally, changes in the funding environment over the period of data collection may have had an effect. National efforts using data from all 50 KL2 programs are underway, and should provide additional insight into differences between the KL2 and the other translational science K-programs.

In conclusion, the research productivity across groups of K-scholars at our institution is broadly similar as are their receipt of R-awards and professional advancement. Furthermore, there is no evidence of increased interdisciplinarity in the research produced by KL2 scholars compared with other types of K-awardees. Additional training directed toward encouraging interdisciplinary and collaborative “team science” during training programs may be needed to supply researchers with the skills needed to address complex biomedical research issues both within and between traditional scientific disciplines.

### Study Highlights

K-scholars’ research productivity and career development are similar between institutional KL2 and individual K-awards. There is no evidence of differing degrees of interdisciplinary research between K-programs. A substantial proportion of K-scholars obtain NIH funding and progress to the rank of Associate Professor, and a large majority continues in research in academic settings.
